# Supplementary material for: Estradiol-induced immune suppression via prostaglandin E2 during parturition in bovine leukemia virus-infected cattle
Source: PLoS One. 2022 Mar 9;17(3):e0263660. doi: 10.1371/journal.pone.0263660 (PMC8906636; doi:10.1371/journal.pone.0263660)
Supplement: S1 Fig — (a) The concentrations of PGE2 in the sera were determined by ELISA. (b and c) Whole-blood culture or PBMC culture was performed to evaluate IFN-γ production in response to Con A or gp51 peptide mix, respectively. (d) The concentrations of estradiol in the sera were determined by ELISA. (PPTX) [file pone.0263660.s002.pptx]

## Slide 1
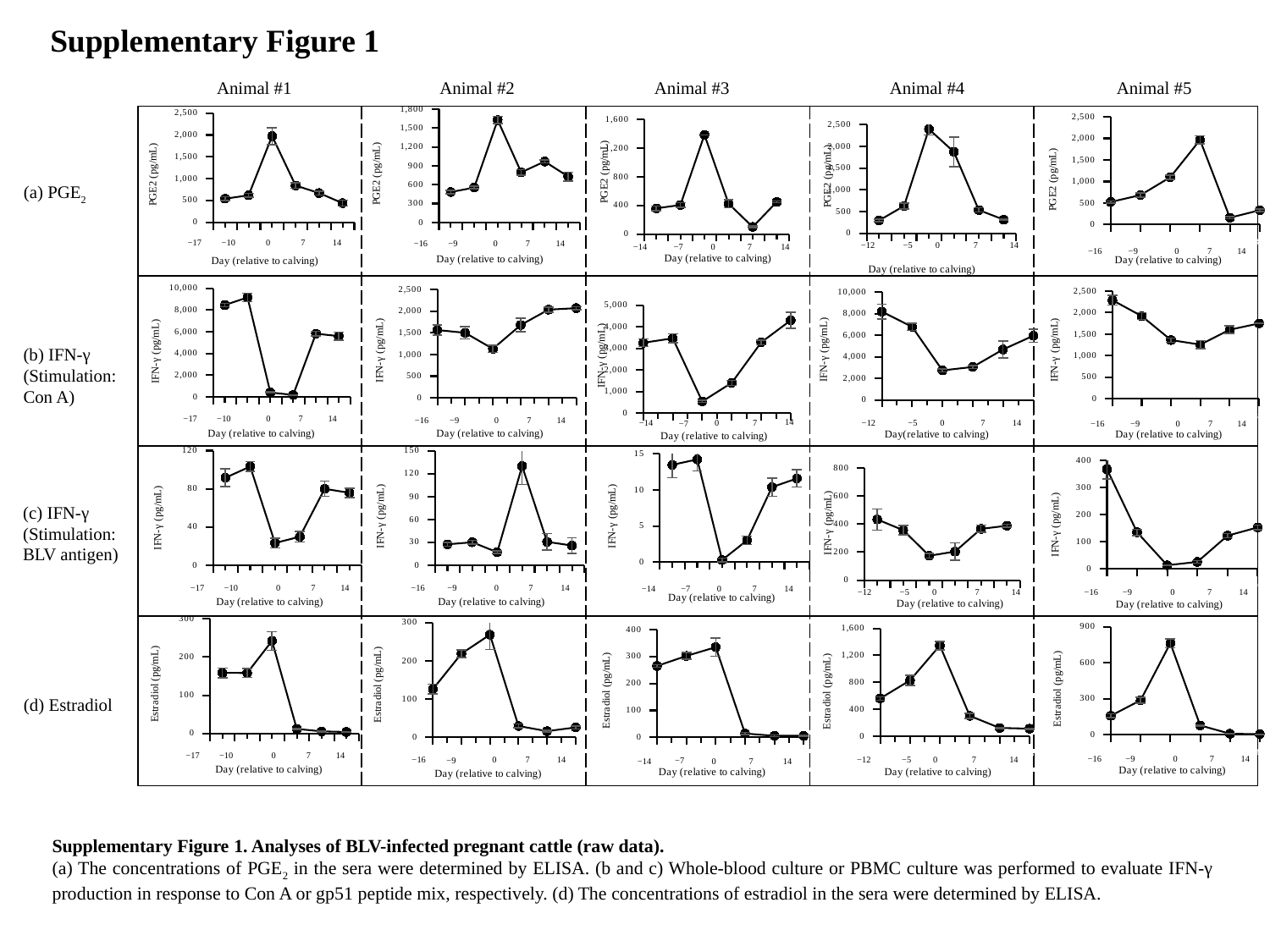

Supplementary Figure 1
Animal #2
Animal #4
Animal #1
Animal #3
Animal #5
### Chart
| Category | PGE2 |
|---|---|
| 0 | 482.6133333333334 |
| 7 | 553.5756913819482 |
| 16 | 1627.5528639442882 |
| 17 | 796.0401591324145 |
| 23 | 968.1735328393887 |
| 30 | 728.9220287529721 |−16
−9
0
7
14
### Chart
| Category | |
|---|---|
| 0 | 1564.7123333333336 |
| 7 | 1500.0656666666666 |
| 16 | 1131.386 |
| 17 | 1680.186 |
| 23 | 2034.647 |
| 30 | 2069.887 |−16
−9
0
7
14
### Chart
| Category | |
|---|---|
| 0 | 27.380666666666666 |
| 7 | 30.100666666666665 |
| 16 | 17.069666666666667 |
| 17 | 129.90566666666666 |
| 23 | 30.647666666666666 |
| 30 | 25.72866666666667 |−9
−16
0
7
14
### Chart
| Category | |
|---|---|
| 0 | 126.0400866251885 |
| 7 | 218.9926705604372 |
| 16 | 268.4888005560413 |
| 17 | 29.231763340053618 |
| 23 | 15.818799331958155 |
| 30 | 25.621602654925812 |−16
0
7
14
−9
### Chart
| Category | PGE2 |
|---|---|
| 0 | 358.72333333333336 |
| 7 | 405.91654807272306 |
| 14 | 1387.67467552445 |
| 15 | 423.4480012102367 |
| 21 | 98.72604819323692 |
| 28 | 449.5454559021261 |−14
−7
0
7
14
### Chart
| Category | PGE2 |
|---|---|
| 0 | 540.967069918013 |
| 7 | 619.4923389984383 |
| 17 | 1974.1886388880896 |
| 18 | 843.3585562065497 |
| 24 | 670.2470059037018 |
| 31 | 439.0792117824167 |−10
0
7
14
−17
| | | | | |
| --- | --- | --- | --- | --- |
| | | | | |
| | | | | |
| | | | | |
### Chart
| Category | |
|---|---|
| 0 | 524.7533333333333 |
| 7 | 684.966039470515 |
| 16 | 1098.6572959340706 |
| 17 | 1963.0015992837627 |
| 23 | 159.78475336905146 |
| 30 | 332.16446348836234 |−9
−16
0
7
14
### Chart
| Category | PGE2 |
|---|---|
| 0 | 295.8664248187273 |
| 7 | 627.3818432518525 |
| 12 | 2393.511177594842 |
| 13 | 1871.2659157520072 |
| 19 | 534.4315125448455 |
| 26 | 313.77348876417983 |−12
−5
0
7
14
(a) PGE2
### Chart
| Category | |
|---|---|
| 0 | 8454.738666666666 |
| 7 | 9155.072 |
| 17 | 417.041 |
| 18 | 190.32966666666667 |
| 24 | 5825.6410000000005 |
| 31 | 5597.621666666666 |−17
−10
0
7
14
### Chart
| Category | |
|---|---|
| 0 | 2288.5499999999997 |
| 7 | 1919.75 |
| 16 | 1363.888 |
| 17 | 1258.0243333333335 |
| 23 | 1603.8239999999998 |
| 30 | 1750.0966666666666 |−9
−16
0
7
14
### Chart
| Category | |
|---|---|
| 0 | 8181.355666666667 |
| 7 | 6771.803666666667 |
| 12 | 2739.0696666666663 |
| 13 | 3056.6766666666663 |
| 19 | 4673.8623333333335 |
| 26 | 5946.888 |−12
−5
0
7
14
### Chart
| Category | |
|---|---|
| 0 | 3262.781333333334 |
| 7 | 3468.8941666666665 |
| 14 | 540.0041666666666 |
| 15 | 1402.4733333333334 |
| 21 | 3283.5066666666667 |
| 28 | 4304.275833333334 |14
−14
0
7
−7
(b) IFN-γ
(Stimulation: Con A)
### Chart
| Category | |
|---|---|
| 0 | 91.529 |
| 7 | 103.26366666666667 |
| 17 | 23.093333333333334 |
| 18 | 29.719000000000005 |
| 24 | 79.98633333333333 |
| 31 | 75.74966666666667 |7
14
−17
−10
0
### Chart
| Category | |
|---|---|
| 0 | 13.460999999999999 |
| 7 | 14.207 |
| 14 | 0.3 |
| 15 | 3.0203333333333333 |
| 21 | 10.383333333333333 |
| 28 | 11.571333333333333 |−14
−7
0
7
14
### Chart
| Category | |
|---|---|
| 0 | 368.2833333333333 |
| 7 | 134.33666666666667 |
| 16 | 11.796666666666667 |
| 17 | 24.188333333333333 |
| 23 | 121.77233333333334 |
| 30 | 152.33833333333334 |−9
−16
0
7
14
### Chart
| Category | |
|---|---|
| 0 | 432.4506666666667 |
| 7 | 356.1933333333333 |
| 12 | 174.0136666666667 |
| 13 | 203.77533333333335 |
| 19 | 365.549 |
| 26 | 387.97433333333333 |−12
−5
0
7
14
(c) IFN-γ
(Stimulation: BLV antigen)
### Chart
| Category | |
|---|---|
| 0 | 158.54735349033473 |
| 7 | 158.40527510672158 |
| 17 | 242.40221803075246 |
| 18 | 11.70647605823003 |
| 24 | 5.2496931348350975 |
| 31 | 3.7461943773010575 |7
14
−17
−10
0
### Chart
| Category | |
|---|---|
| 0 | 157.03666666666666 |
| 7 | 287.19353351556305 |
| 16 | 763.2333333333332 |
| 17 | 76.75848383506205 |
| 23 | 7.358925348865742 |
| 30 | 3.4742097807972754 |−9
−16
0
7
14
### Chart
| Category | |
|---|---|
| 0 | 559.8986023902598 |
| 7 | 827.8995259899467 |
| 12 | 1345.776975327225 |
| 13 | 301.7211460059754 |
| 19 | 122.23878973257007 |
| 26 | 111.0719255038454 |−12
−5
0
7
14
### Chart
| Category | |
|---|---|
| 0 | 264.94666666666666 |
| 7 | 301.9857808108687 |
| 14 | 334.5872995619047 |
| 15 | 14.090182959731974 |
| 21 | 5.477202513030634 |
| 28 | 5.407431318102813 |−7
−14
0
7
14
(d) Estradiol
Supplementary Figure 1. Analyses of BLV-infected pregnant cattle (raw data).
(a) The concentrations of PGE2 in the sera were determined by ELISA. (b and c) Whole-blood culture or PBMC culture was performed to evaluate IFN-γ production in response to Con A or gp51 peptide mix, respectively. (d) The concentrations of estradiol in the sera were determined by ELISA.
