# Supplementary material for: Estradiol-induced immune suppression via prostaglandin E2 during parturition in bovine leukemia virus-infected cattle
Source: PLoS One. 2022 Mar 9;17(3):e0263660. doi: 10.1371/journal.pone.0263660 (PMC8906636; doi:10.1371/journal.pone.0263660)
Supplement: S2 Fig — (a–d) Estradiol administration was performed using BLV-infected cattle (animals #6–#10). (a) Serum estradiol concentrations were measured by ELISA. (b) Serum PGE2 concentrations were measured by ELISA. (c and d) Whole-blood culture or PBMC culture was performed to evaluate IFN-γ production in response to Con A or gp51 peptide mix, respectively. (PPTX) [file pone.0263660.s003.pptx]

## Slide 1
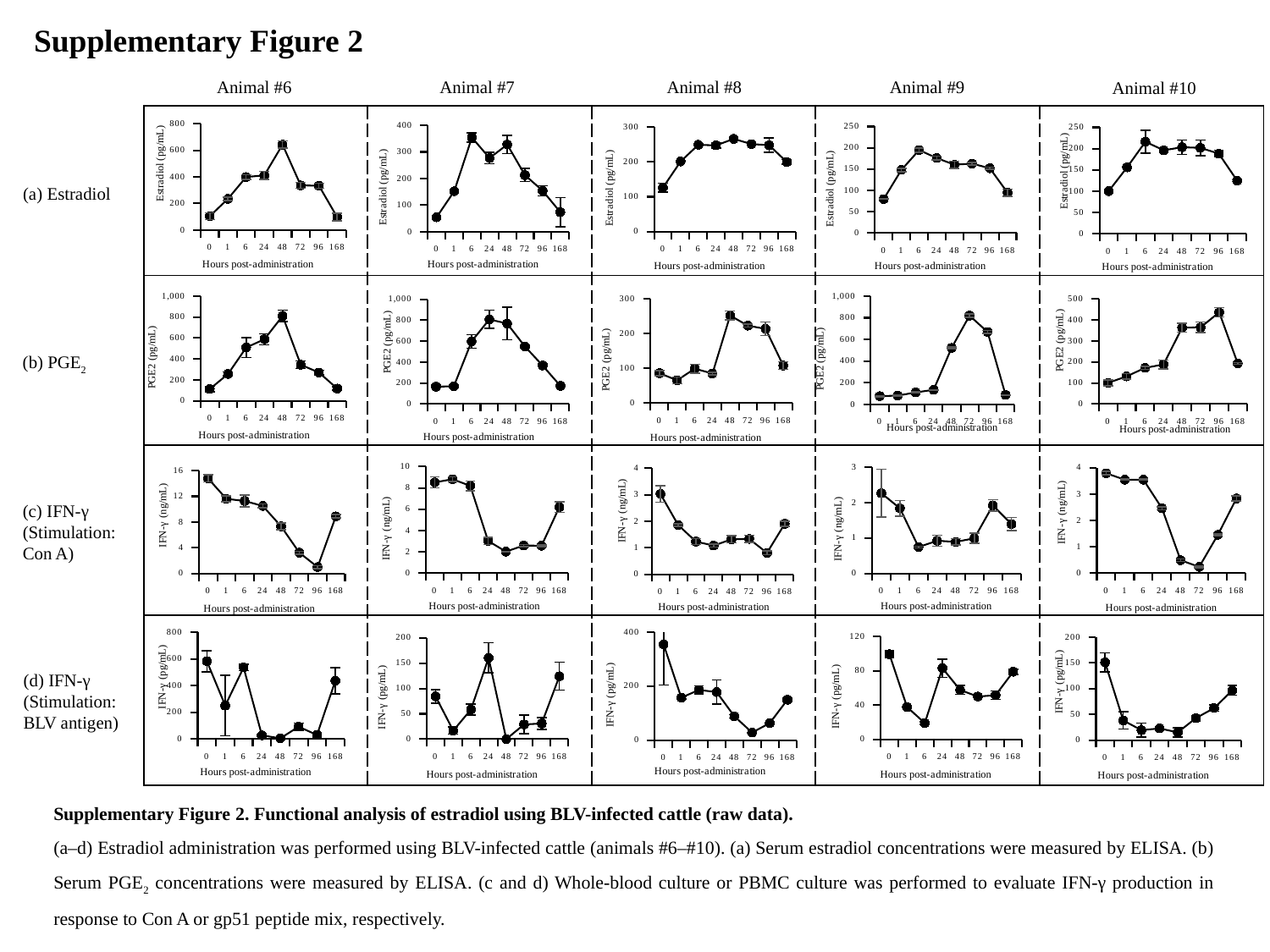

Supplementary Figure 2
Animal #7
Animal #9
Animal #6
Animal #8
Animal #10
### Chart
| Category | |
|---|---|
| 0 | 103.68760186469429 |
| 1 | 234.06275862864211 |
| 6 | 398.5103500000852 |
| 24 | 411.17763420831653 |
| 48 | 642.8017063318083 |
| 72 | 335.39984379114145 |
| 96 | 333.1922912850168 |
| 168 | 96.7205980140743 || | | | | |
| --- | --- | --- | --- | --- |
| | | | | |
| | | | | |
| | | | | |
### Chart
| Category | |
|---|---|
| 0 | 54.1873268061643 |
| 1 | 152.04147548413297 |
| 6 | 354.00593702439727 |
| 24 | 276.7024052550391 |
| 48 | 327.50368617405223 |
| 72 | 212.7151798076101 |
| 96 | 153.89668488491807 |
| 168 | 73.44179120440249 |
### Chart
| Category | |
|---|---|
| 0 | 79.58485122924489 |
| 1 | 147.90347422940096 |
| 6 | 194.44235016355304 |
| 24 | 175.77017862803274 |
| 48 | 160.10196530463165 |
| 72 | 162.73504689970707 |
| 96 | 151.82422988133754 |
| 168 | 94.42381487632477 |
### Chart
| Category | |
|---|---|
| 0 | 126.23332509709073 |
| 1 | 200.98180083643908 |
| 6 | 248.89459623373403 |
| 24 | 246.98887747567667 |
| 48 | 265.6676052085363 |
| 72 | 250.92584433972252 |
| 96 | 247.64875533648183 |
| 168 | 199.40742808987596 |
### Chart
| Category | |
|---|---|
| 0 | 100.11760278174336 |
| 1 | 155.8330755091065 |
| 6 | 216.2184568403749 |
| 24 | 196.0421288298656 |
| 48 | 203.26497129005034 |
| 72 | 201.71004399785372 |
| 96 | 188.130746028896 |
| 168 | 124.64511836948881 |(a) Estradiol
### Chart
| Category | |
|---|---|
| 0 | 75.23921889120152 |
| 1 | 80.49019505192788 |
| 6 | 109.74103026409765 |
| 24 | 134.258637796218 |
| 48 | 521.6113293192668 |
| 72 | 819.4104899883039 |
| 96 | 669.0459906736356 |
| 168 | 87.11305372213229 |
### Chart
| Category | |
|---|---|
| 0 | 112.61102149989722 |
| 1 | 256.3131587609691 |
| 6 | 508.79768629493213 |
| 24 | 587.1663214679556 |
| 48 | 809.7386481509226 |
| 72 | 344.28752857544004 |
| 96 | 269.81789058827377 |
| 168 | 116.93200887565682 |
### Chart
| Category | |
|---|---|
| 0 | 165.3809929066953 |
| 1 | 169.29012370985788 |
| 6 | 597.1636986635607 |
| 24 | 807.7877570747487 |
| 48 | 768.4047232241852 |
| 72 | 549.6554776912 |
| 96 | 367.6575145573129 |
| 168 | 173.45988521385854 |
### Chart
| Category | |
|---|---|
| 0 | 86.11942833710593 |
| 1 | 65.62417565353113 |
| 6 | 99.06610562901326 |
| 24 | 84.90777754737546 |
| 48 | 251.80571721337088 |
| 72 | 222.55044330017304 |
| 96 | 213.47384720362516 |
| 168 | 108.11593404249115 |
### Chart
| Category | |
|---|---|
| 0 | 100.3406701190427 |
| 1 | 131.15115908021158 |
| 6 | 171.45455063330567 |
| 24 | 188.16918280193525 |
| 48 | 362.4555026224666 |
| 72 | 364.26338376042503 |
| 96 | 435.4730437634799 |
| 168 | 192.63633631798908 |(b) PGE2
### Chart
| Category | |
|---|---|
| 0 | 8.516666666666667 |
| 1 | 8.816666666666668 |
| 6 | 8.19 |
| 24 | 3.0199999999999996 |
| 48 | 2.013333333333333 |
| 72 | 2.586666666666667 |
| 96 | 2.5700000000000003 |
| 168 | 6.19 |
### Chart
| Category | |
|---|---|
| 0 | 2.268025 |
| 1 | 1.8392970000000002 |
| 6 | 0.7451053333333334 |
| 24 | 0.9224663333333334 |
| 48 | 0.892185 |
| 72 | 0.9919546666666667 |
| 96 | 1.9225446666666663 |
| 168 | 1.393769 |
### Chart
| Category | |
|---|---|
| 0 | 3.7887376666666666 |
| 1 | 3.544726666666667 |
| 6 | 3.544726666666667 |
| 24 | 2.4613346666666667 |
| 48 | 0.4909296666666667 |
| 72 | 0.23723066666666667 |
| 96 | 1.4522313333333334 |
| 168 | 2.8308366666666664 |
### Chart
| Category | |
|---|---|
| 0 | 3.0277396666666667 |
| 1 | 1.8546446666666665 |
| 6 | 1.2308000000000001 |
| 24 | 1.0792303333333333 |
| 48 | 1.3173453333333336 |
| 72 | 1.3318786666666667 |
| 96 | 0.8077833333333334 |
| 168 | 1.9002813333333333 |
### Chart
| Category | |
|---|---|
| 0 | 14.766666666666666 |
| 1 | 11.6 |
| 6 | 11.266666666666666 |
| 24 | 10.5 |
| 48 | 7.326666666666667 |
| 72 | 3.23 |
| 96 | 0.9936666666666668 |
| 168 | 8.883333333333333 |(c) IFN-γ
(Stimulation: Con A)
### Chart
| Category | |
|---|---|
| 0 | 354.08000000000004 |
| 1 | 157.29500000000002 |
| 6 | 185.788 |
| 24 | 177.894 |
| 48 | 88.44399999999999 |
| 72 | 28.399 |
| 96 | 63.2875 |
| 168 | 149.5415 |
### Chart
| Category | |
|---|---|
| 0 | 582.8776666666666 |
| 1 | 249.61733333333333 |
| 6 | 535.7396666666667 |
| 24 | 26.439666666666668 |
| 48 | 3.975 |
| 72 | 91.18066666666668 |
| 96 | 29.200333333333333 |
| 168 | 436.90766666666667 |
### Chart
| Category | |
|---|---|
| 0 | 99.25999999999999 |
| 1 | 37.6415 |
| 6 | 18.991500000000002 |
| 24 | 83.02000000000001 |
| 48 | 57.6295 |
| 72 | 49.935 |
| 96 | 51.573499999999996 |
| 168 | 78.8415 |
### Chart
| Category | |
|---|---|
| 0 | 150.904 |
| 1 | 38.048 |
| 6 | 19.285666666666668 |
| 24 | 22.672666666666668 |
| 48 | 15.020666666666665 |
| 72 | 42.68866666666667 |
| 96 | 62.19499999999999 |
| 168 | 96.63166666666666 |
### Chart
| Category | |
|---|---|
| 0 | 84.52933333333333 |
| 1 | 16.672666666666668 |
| 6 | 58.38666666666666 |
| 24 | 160.60266666666666 |
| 48 | 0.0 |
| 72 | 28.917333333333335 |
| 96 | 30.799666666666667 |
| 168 | 124.20333333333333 |(d) IFN-γ
(Stimulation: BLV antigen)
Supplementary Figure 2. Functional analysis of estradiol using BLV-infected cattle (raw data).
(a–d) Estradiol administration was performed using BLV-infected cattle (animals #6–#10). (a) Serum estradiol concentrations were measured by ELISA. (b) Serum PGE2 concentrations were measured by ELISA. (c and d) Whole-blood culture or PBMC culture was performed to evaluate IFN-γ production in response to Con A or gp51 peptide mix, respectively.
