# Supplementary material for: Estradiol-induced immune suppression via prostaglandin E2 during parturition in bovine leukemia virus-infected cattle
Source: PLoS One. 2022 Mar 9;17(3):e0263660. doi: 10.1371/journal.pone.0263660 (PMC8906636; doi:10.1371/journal.pone.0263660)
Supplement: S1 Table — (PPTX) [file pone.0263660.s004.pptx]

## Slide 1
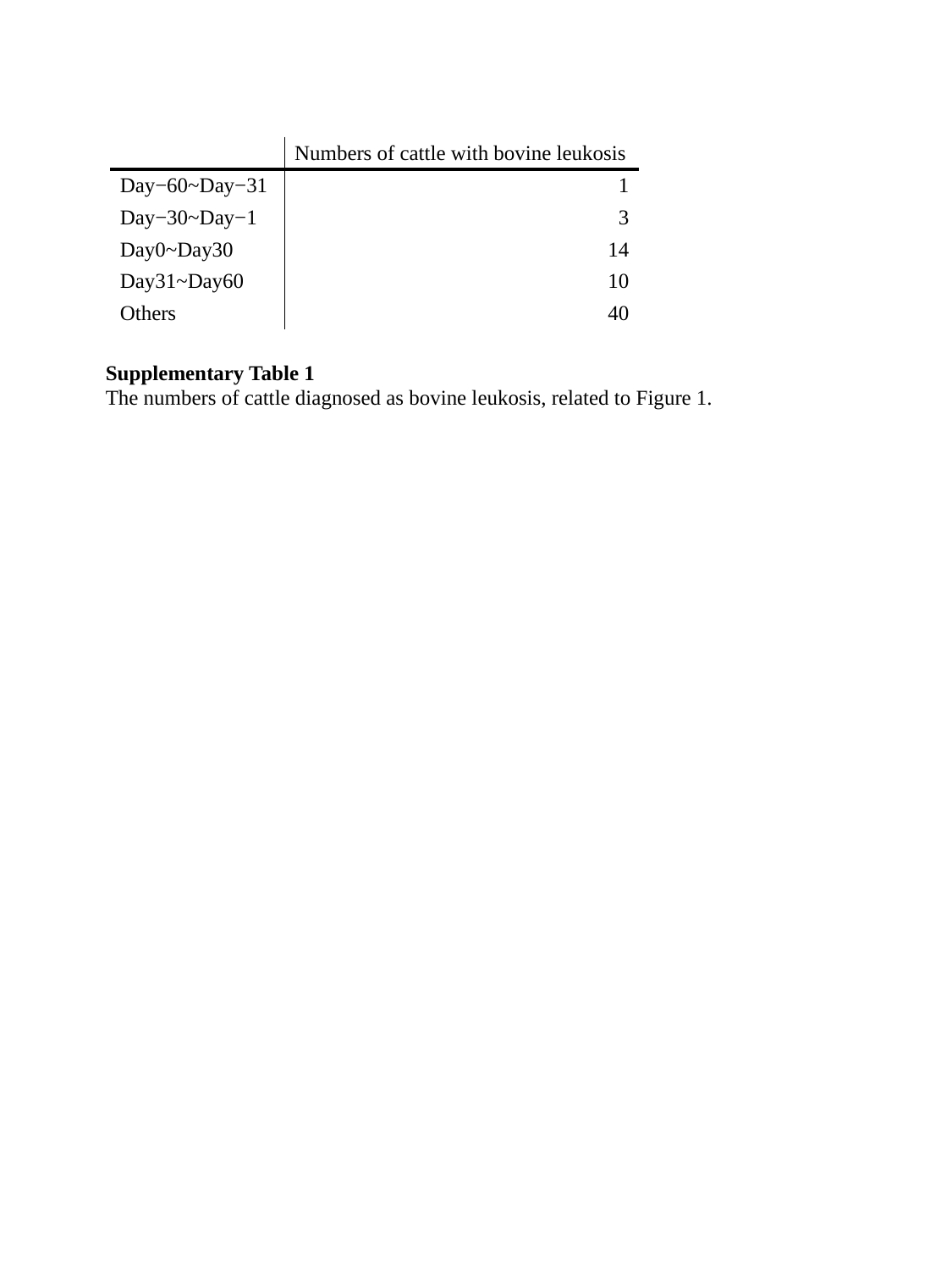

| | Numbers of cattle with bovine leukosis |
| --- | --- |
| Day−60~Day−31 | 1 |
| Day−30~Day−1 | 3 |
| Day0~Day30 | 14 |
| Day31~Day60 | 10 |
| Others | 40 |
Supplementary Table 1
The numbers of cattle diagnosed as bovine leukosis, related to Figure 1.
