# Supplementary material for: Estradiol-induced immune suppression via prostaglandin E2 during parturition in bovine leukemia virus-infected cattle
Source: PLoS One. 2022 Mar 9;17(3):e0263660. doi: 10.1371/journal.pone.0263660 (PMC8906636; doi:10.1371/journal.pone.0263660)
Supplement: S2 Table — (a) The concentrations of PGE2 in the sera. (b and c) IFN-γ production in response to Con A (b) or gp51 peptide mix (c) in the whole-blood cultures (b) or PBMC cultures (c). (d) The concentrations of estradiol in the sera. (PPTX) [file pone.0263660.s005.pptx]

## Slide 1
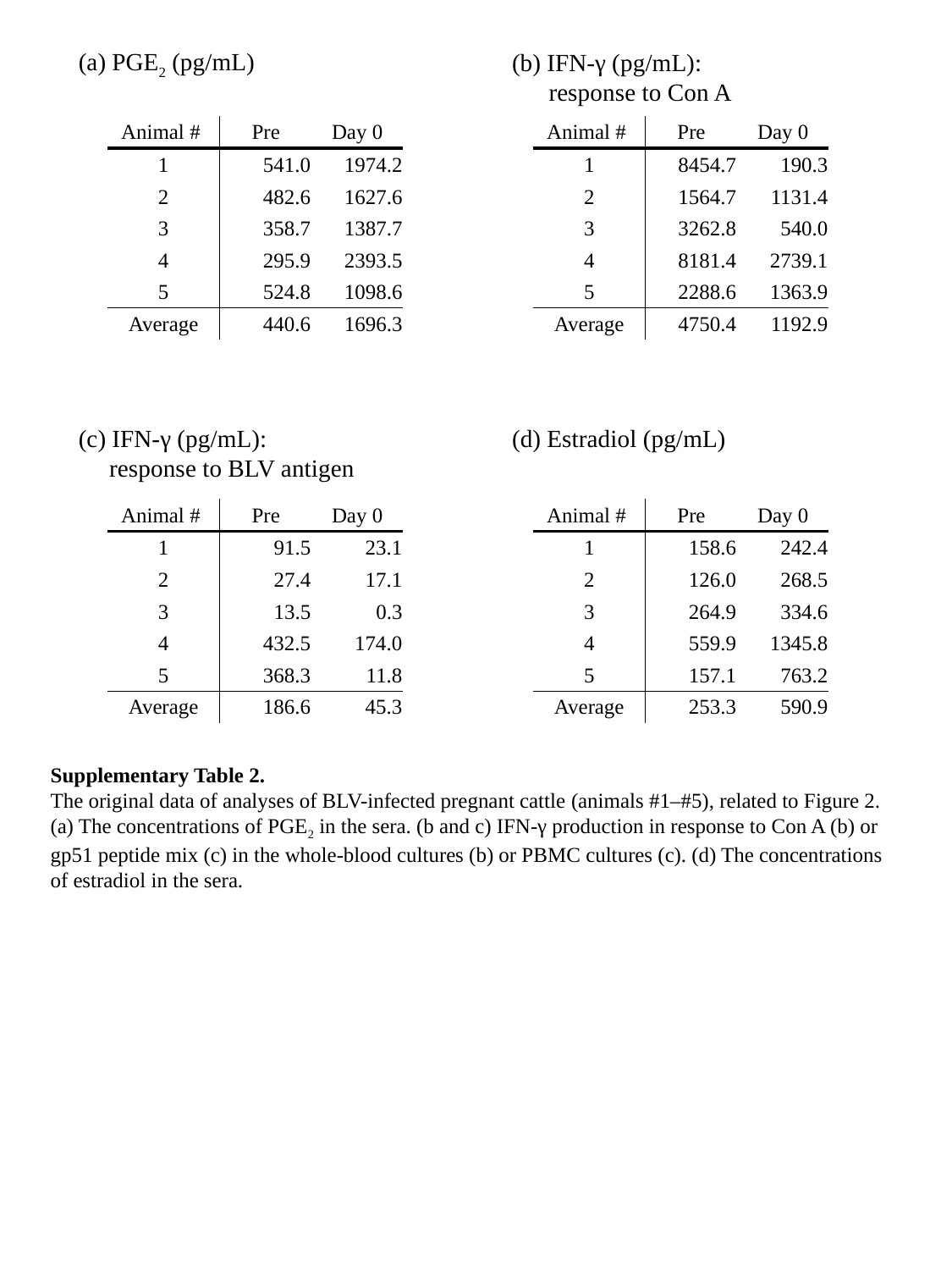

(a) PGE2 (pg/mL)
(b) IFN-γ (pg/mL):
 response to Con A
| Animal # | Pre | Day 0 |
| --- | --- | --- |
| 1 | 541.0 | 1974.2 |
| 2 | 482.6 | 1627.6 |
| 3 | 358.7 | 1387.7 |
| 4 | 295.9 | 2393.5 |
| 5 | 524.8 | 1098.6 |
| Average | 440.6 | 1696.3 |
| Animal # | Pre | Day 0 |
| --- | --- | --- |
| 1 | 8454.7 | 190.3 |
| 2 | 1564.7 | 1131.4 |
| 3 | 3262.8 | 540.0 |
| 4 | 8181.4 | 2739.1 |
| 5 | 2288.6 | 1363.9 |
| Average | 4750.4 | 1192.9 |
(c) IFN-γ (pg/mL):
 response to BLV antigen
(d) Estradiol (pg/mL)
| Animal # | Pre | Day 0 |
| --- | --- | --- |
| 1 | 91.5 | 23.1 |
| 2 | 27.4 | 17.1 |
| 3 | 13.5 | 0.3 |
| 4 | 432.5 | 174.0 |
| 5 | 368.3 | 11.8 |
| Average | 186.6 | 45.3 |
| Animal # | Pre | Day 0 |
| --- | --- | --- |
| 1 | 158.6 | 242.4 |
| 2 | 126.0 | 268.5 |
| 3 | 264.9 | 334.6 |
| 4 | 559.9 | 1345.8 |
| 5 | 157.1 | 763.2 |
| Average | 253.3 | 590.9 |
Supplementary Table 2.
The original data of analyses of BLV-infected pregnant cattle (animals #1–#5), related to Figure 2.
(a) The concentrations of PGE2 in the sera. (b and c) IFN-γ production in response to Con A (b) or gp51 peptide mix (c) in the whole-blood cultures (b) or PBMC cultures (c). (d) The concentrations of estradiol in the sera.
