# Supplementary material for: Estradiol-induced immune suppression via prostaglandin E2 during parturition in bovine leukemia virus-infected cattle
Source: PLoS One. 2022 Mar 9;17(3):e0263660. doi: 10.1371/journal.pone.0263660 (PMC8906636; doi:10.1371/journal.pone.0263660)
Supplement: S3 Table — (a and b) IFN-γ production in the cultures of PBMCs from BLV-uninfected (a) and infected cattle (b) cultivated with anti-CD3 and anti-CD28 mAbs (a) and FLK-BLV (b), respectively. (c and d) COX2 expression (c) and PGE2 concentration (d) in PBMCs from BLV-uninfected cattle cultivated with estradiol. (e) IFN-γ production in PBMCs from BLV-uninfected cattle cultivated with each EP antagonist, estradiol, and anti-CD3 and anti-CD28 mAbs. (PPTX) [file pone.0263660.s006.pptx]

## Slide 1
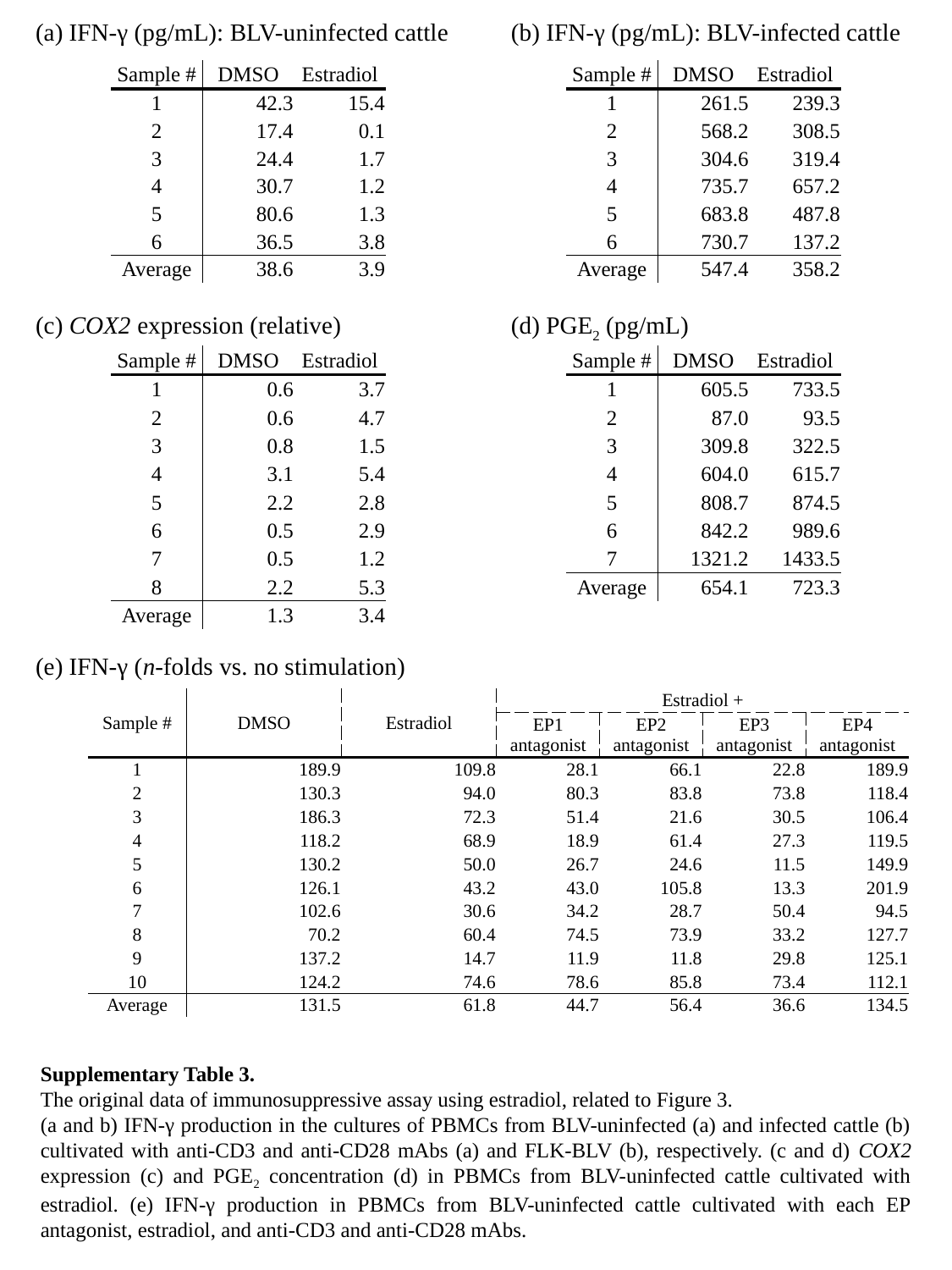

(a) IFN-γ (pg/mL): BLV-uninfected cattle
(b) IFN-γ (pg/mL): BLV-infected cattle
| Sample # | DMSO | Estradiol |
| --- | --- | --- |
| 1 | 42.3 | 15.4 |
| 2 | 17.4 | 0.1 |
| 3 | 24.4 | 1.7 |
| 4 | 30.7 | 1.2 |
| 5 | 80.6 | 1.3 |
| 6 | 36.5 | 3.8 |
| Average | 38.6 | 3.9 |
| Sample # | DMSO | Estradiol |
| --- | --- | --- |
| 1 | 261.5 | 239.3 |
| 2 | 568.2 | 308.5 |
| 3 | 304.6 | 319.4 |
| 4 | 735.7 | 657.2 |
| 5 | 683.8 | 487.8 |
| 6 | 730.7 | 137.2 |
| Average | 547.4 | 358.2 |
(c) COX2 expression (relative)
(d) PGE2 (pg/mL)
| Sample # | DMSO | Estradiol |
| --- | --- | --- |
| 1 | 0.6 | 3.7 |
| 2 | 0.6 | 4.7 |
| 3 | 0.8 | 1.5 |
| 4 | 3.1 | 5.4 |
| 5 | 2.2 | 2.8 |
| 6 | 0.5 | 2.9 |
| 7 | 0.5 | 1.2 |
| 8 | 2.2 | 5.3 |
| Average | 1.3 | 3.4 |
| Sample # | DMSO | Estradiol |
| --- | --- | --- |
| 1 | 605.5 | 733.5 |
| 2 | 87.0 | 93.5 |
| 3 | 309.8 | 322.5 |
| 4 | 604.0 | 615.7 |
| 5 | 808.7 | 874.5 |
| 6 | 842.2 | 989.6 |
| 7 | 1321.2 | 1433.5 |
| Average | 654.1 | 723.3 |
(e) IFN-γ (n-folds vs. no stimulation)
| Sample # | DMSO | Estradiol | Estradiol + | | | |
| --- | --- | --- | --- | --- | --- | --- |
| | | | EP1 antagonist | EP2 antagonist | EP3 antagonist | EP4 antagonist |
| 1 | 189.9 | 109.8 | 28.1 | 66.1 | 22.8 | 189.9 |
| 2 | 130.3 | 94.0 | 80.3 | 83.8 | 73.8 | 118.4 |
| 3 | 186.3 | 72.3 | 51.4 | 21.6 | 30.5 | 106.4 |
| 4 | 118.2 | 68.9 | 18.9 | 61.4 | 27.3 | 119.5 |
| 5 | 130.2 | 50.0 | 26.7 | 24.6 | 11.5 | 149.9 |
| 6 | 126.1 | 43.2 | 43.0 | 105.8 | 13.3 | 201.9 |
| 7 | 102.6 | 30.6 | 34.2 | 28.7 | 50.4 | 94.5 |
| 8 | 70.2 | 60.4 | 74.5 | 73.9 | 33.2 | 127.7 |
| 9 | 137.2 | 14.7 | 11.9 | 11.8 | 29.8 | 125.1 |
| 10 | 124.2 | 74.6 | 78.6 | 85.8 | 73.4 | 112.1 |
| Average | 131.5 | 61.8 | 44.7 | 56.4 | 36.6 | 134.5 |
Supplementary Table 3.
The original data of immunosuppressive assay using estradiol, related to Figure 3.
(a and b) IFN-γ production in the cultures of PBMCs from BLV-uninfected (a) and infected cattle (b) cultivated with anti-CD3 and anti-CD28 mAbs (a) and FLK-BLV (b), respectively. (c and d) COX2 expression (c) and PGE2 concentration (d) in PBMCs from BLV-uninfected cattle cultivated with estradiol. (e) IFN-γ production in PBMCs from BLV-uninfected cattle cultivated with each EP antagonist, estradiol, and anti-CD3 and anti-CD28 mAbs.
