# Supplementary material for: Estradiol-induced immune suppression via prostaglandin E2 during parturition in bovine leukemia virus-infected cattle
Source: PLoS One. 2022 Mar 9;17(3):e0263660. doi: 10.1371/journal.pone.0263660 (PMC8906636; doi:10.1371/journal.pone.0263660)
Supplement: S4 Table — (a) The concentrations of estradiol in the sera. (b) The concentrations of PGE2 in the sera. (c and d) IFN-γ production in response to Con A (c) or gp51 peptide mix (d) in the whole-blood cultures (c) or PBMC cultures (d). (PPTX) [file pone.0263660.s007.pptx]

## Slide 1
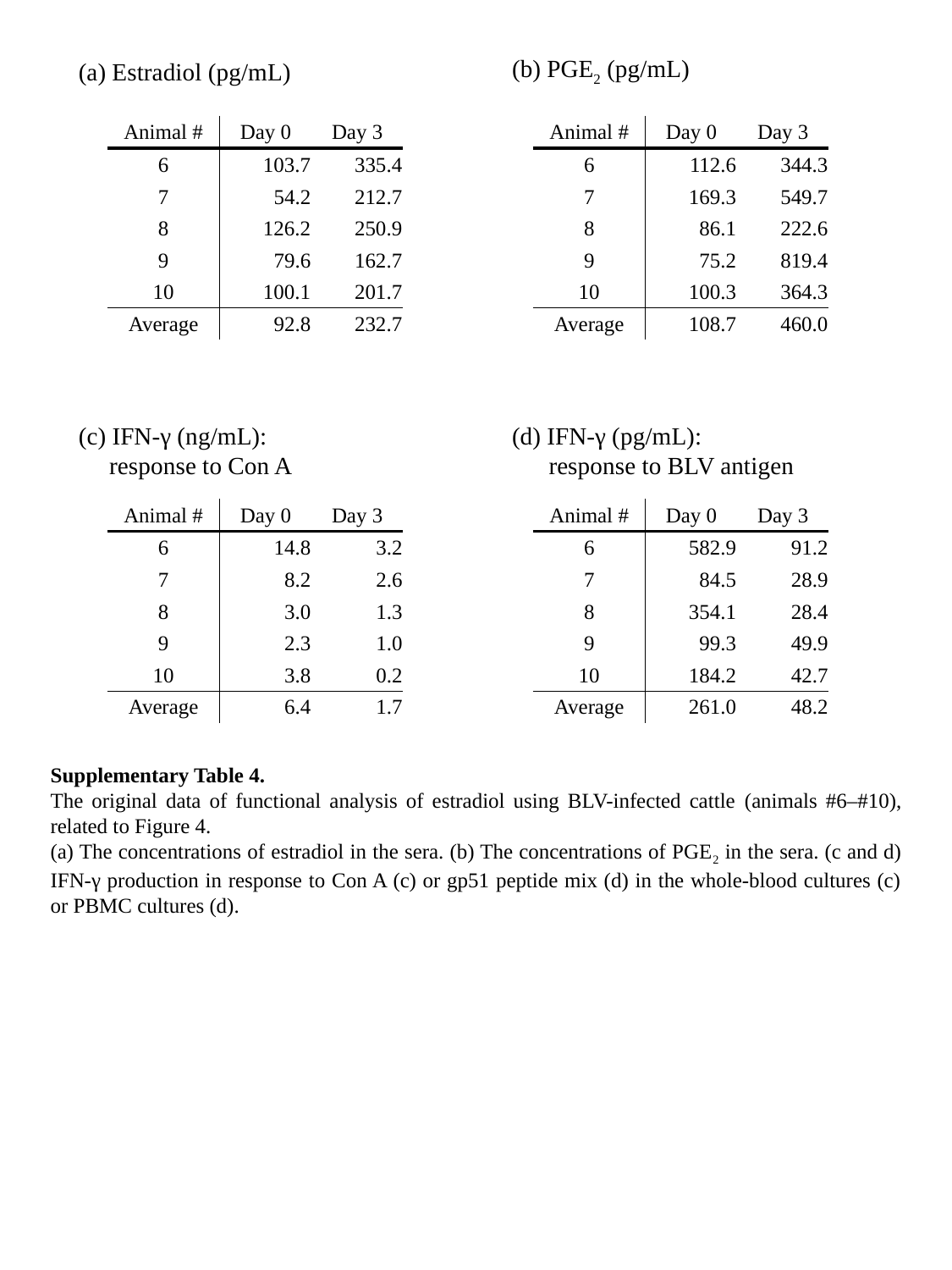

(b) PGE2 (pg/mL)
(a) Estradiol (pg/mL)
| Animal # | Day 0 | Day 3 |
| --- | --- | --- |
| 6 | 103.7 | 335.4 |
| 7 | 54.2 | 212.7 |
| 8 | 126.2 | 250.9 |
| 9 | 79.6 | 162.7 |
| 10 | 100.1 | 201.7 |
| Average | 92.8 | 232.7 |
| Animal # | Day 0 | Day 3 |
| --- | --- | --- |
| 6 | 112.6 | 344.3 |
| 7 | 169.3 | 549.7 |
| 8 | 86.1 | 222.6 |
| 9 | 75.2 | 819.4 |
| 10 | 100.3 | 364.3 |
| Average | 108.7 | 460.0 |
(c) IFN-γ (ng/mL):
 response to Con A
(d) IFN-γ (pg/mL):
 response to BLV antigen
| Animal # | Day 0 | Day 3 |
| --- | --- | --- |
| 6 | 14.8 | 3.2 |
| 7 | 8.2 | 2.6 |
| 8 | 3.0 | 1.3 |
| 9 | 2.3 | 1.0 |
| 10 | 3.8 | 0.2 |
| Average | 6.4 | 1.7 |
| Animal # | Day 0 | Day 3 |
| --- | --- | --- |
| 6 | 582.9 | 91.2 |
| 7 | 84.5 | 28.9 |
| 8 | 354.1 | 28.4 |
| 9 | 99.3 | 49.9 |
| 10 | 184.2 | 42.7 |
| Average | 261.0 | 48.2 |
Supplementary Table 4.
The original data of functional analysis of estradiol using BLV-infected cattle (animals #6–#10), related to Figure 4.
(a) The concentrations of estradiol in the sera. (b) The concentrations of PGE2 in the sera. (c and d) IFN-γ production in response to Con A (c) or gp51 peptide mix (d) in the whole-blood cultures (c) or PBMC cultures (d).
