# Supplementary material for: Estradiol-induced immune suppression via prostaglandin E2 during parturition in bovine leukemia virus-infected cattle
Source: PLoS One. 2022 Mar 9;17(3):e0263660. doi: 10.1371/journal.pone.0263660 (PMC8906636; doi:10.1371/journal.pone.0263660)
Supplement: Attachment — Submitted filename: Response to reviewers.docx [file pone.0263660.s008.docx]

Manuscript ID : PONE-D-21-17024

Editor in PLOS ONE

Prof. Juan J Loor

December 15, 2021

Dear Prof. Juan J Loor

I wish to submit electronically, a revised version of the manuscript (1^st^ revision) entitled “Estradiol - induced immune suppression via prostaglandin E_2_ during parturition in bovine leukemia virus-infected cattle” by Yamato Sajiki, Satoru Konnai, Tomohiro Okagawa, Naoya Maekawa, Shinya Goto, Junko Kohara, Atsushi Nitanai, Hirofumi Takahashi, Kentaro Kubota, Hiroshi Takeda, Shiro Murata, Kazuhiko Ohashi, which I would like to submit for publication in PLOS ONE.

Regarding comments of the reviewer#1, we amended our first revised manuscript as follows.

Attached herewith is the revised manuscript (highlighted) and Figures.

Your kind cooperation would be gratefully appreciated.

Sincerely yours,

Satoru KONNAI

Department of Disease Control,

Faculty of Veterinary Med.,

Hokkaido University

Sapporo 060-0818, Japan

Tel, +81-11-706-5216

FAX, +81-11-706-5217

E-mail, konnai@vetmed.hokudai.ac.jp

Answers to the comments of the Reviewer #1

Thank you very much for your comments. We appreciate your comments and amended our manuscript accordingly.

Answers to the comments are as follows:

Reviewer#1s' comments:

This manuscript addresses a very important need for better definition of factors that impact immunosuppression in the peripartum period. The focus on PGE2 and estradiol is an important consideration and has yielded insight on specific receptor involvement. However, it is unclear if the focus is on at the time of parturition or the surrounding period? The outcomes that determined specific conclusion on the selected term eg effect at parturition for the observed effects are not clear. More information on age, stage of lactation, parity, breed, diet and management etc would help uncover other possible contributing factors and should at least be discussed. Furthermore, the possible contribution of declining progesterone concentration (known immunosuppressant) and health parameters such status of infection by other pathogens, occurrence of diseases such as laminitis, mastitis etc. are not considered. In light of the impact of LPS on in vitro COX2 expression and PGE2 secretion what were the levels of endotoxins in reagents and containers for in vitro studies and what controls were used to evaluate the possible contribution of LPS? Discuss the use of the DMSO control versus buffer or known plasma. Please check sentence structure/missing words etc. eg line 2 and line 12.1, Not clear what this sentence means please edit Line 2 In dairy cattle, efficient milk production continues to require for pregnancy and 3 parturition each year.

1. **‘**It is unclear if the focus is on at the time of parturition or the surrounding period?. The outcomes that determined specific conclusion on the selected term eg effect at parturition for the observed effects are not clear**’**

**Ans:** Immune suppression during pregnancy and parturition is regarded as a risk factor related to the progression of bovine chronic infections, such as bovine leukemia virus (BLV) infection. In our previous study, we demonstrated that prostaglandin E_2_ (PGE_2_) suppresses BLV-specific Th1 responses and contributes to the progression of BLV infection (Sajiki et al., *J. Immunol.,* 2019, 203(5):1313-1324.). Thus, we focused on parturition because PGE_2_ and estradiol, which suppress immunity, are secreted by the placenta. This study was performed to investigate the involvement of estradiol-induced PGE_2_ in immune suppression during parturition. We believe that our study makes a significant contribution to the literature as a translational medical research because our data suggest that PGE_2_ upregulation inhibits Th1 responses during parturition. Additionally, estradiol was essential for PGE_2_ induction in pregnant cattle. Taken together, our data suggest that, in pregnant cattle, estradiol-induced PGE_2_ is involved in the suppression of Th1 responses, which contributes to the progression of bovine chronic infections.

2. **‘**More information on age, stage of lactation, parity, breed, diet and management etc would help uncover other possible contributing factors and should at least be discussed**’**

**Ans:** As you pointed out, other factors such as lactation stage, parity, diet and management are also important issues as risk factors for developing leukemia. However, unfortunately, due to various restrictions, the information was not available. In this study, we focused only on parturition because PGE_2_ and estradiol, which suppress immunity, are secreted by the placenta, but we would like to consider other risk factors in the future. We would like to present it as a follow-up report.

3. **‘**the possible contribution of declining progesterone concentration (known immunosuppressant) and health parameters such status of infection by other pathogens, occurrence of diseases such as laminitis, mastitis etc. are not considered**’**

**Ans:** Thank you very much for your interesting comments. This study does not show a causal relationship between the onset of the disease and other health parameters such as infection status by other pathogens or the occurrence of diseases such as laminitis and mastitis, but we believe this is something that we should also focus on. Actually, we are currently investigating these points for implementation, and this is one of our future studies. We would like to present it as a follow-up report.

4. **‘**In light of the impact of LPS on in vitro COX2 expression and PGE2 secretion what were the levels of endotoxins in reagents and containers for in vitro studies and what controls were used to evaluate the possible contribution of LPS?**’**

**Ans:** As you pointed out, LPS is an important factor. To avoid the effects of LPS contamination, we used commercially available endotoxin (LPS)-free regents and containers for this experiment. Therefore, we did not perform any measurements.

5. **‘**Discuss the use of the DMSO control versus buffer or known plasma**’**

**Ans:** PGE_2_ and estradiol are extremely insoluble in water. Therefore, DMSO is used as the best solvent. In this study, DMSO was used as the solvent as in our previous experiments. Since high concentrations of DMSO are cytotoxic, we used the same concentration of DMSO as a negative control.

6. **‘**Please check sentence structure/missing words etc. eg line 2 and line 12.1, Not clear what this sentence means please edit Line 2 In dairy cattle, efficient milk production continues to require for pregnancy and 3 parturition each year**’**

**Ans:** Following your comment, we modified the sentence to ‘In dairy cattle, efficient milk production continues to require for pregnancy and 3 parturition each year’.
